# Dominant Groups of Potentially Active Bacteria Shared by Barley Seeds become Less Abundant in Root Associated Microbiome

**DOI:** 10.3389/fpls.2017.01005

**Published:** 2017-06-15

**Authors:** Luhua Yang, Jasmin Danzberger, Anne Schöler, Peter Schröder, Michael Schloter, Viviane Radl

**Affiliations:** Research Unit, Comparative Microbiome Analysis, Helmholtz Zentrum München,München, Germany

**Keywords:** *Hordeum vulgare* L., root endophytes, seed microbiome, 16S rRNA barcoding

## Abstract

Endophytes are microorganisms colonizing plant internal tissues. They are ubiquitously associated with plants and play an important role in plant growth and health. In this work, we grew five modern cultivars of barley in axenic systems using sterile sand mixture as well as in greenhouse with natural soil. We characterized the potentially active microbial communities associated with seeds and roots using rRNA based amplicon sequencing. The seeds of the different cultivars share a great part of their microbiome, as we observed a predominance of a few bacterial OTUs assigned to *Phyllobacterium*, *Paenibacillus*, and *Trabusiella*. Seed endophytes, particularly members of the Enterobacteriacea and Paenibacillaceae, were important members of root endophytes in axenic systems, where there were no external microbes. However, when plants were grown in soil, seed endophytes became less abundant in root associated microbiome. We observed a clear enrichment of Actinobacteriacea and Rhizobiaceae, indicating a strong influence of the soil bacterial communities on the composition of the root microbiome. Two OTUs assigned to Phyllobacteriaceae were found in all seeds and root samples growing in soil, indicating a relationship between seed-borne and root associated microbiome in barley. Even though the role of endophytic bacteria remains to be clarified, it is known that many members of the genera detected in our study produce phytohormones, shape seedling exudate profile and may play an important role in germination and establishment of the seedlings.

## Introduction

Microorganisms living in close association with plants have significant impact on plant growth and health. Hence, the plant associated microbiome is often referred to as the “second genome” of plants (Berendsen et al., 2012). A special role has been assigned to those communities living inside plant organs for all or part of their lifetime, termed as endophytes (Hardoim et al., 2015). Due to their intimate association with plant tissues (Han et al., 2016), they impact the development of the host significantly (Berg et al., 2016; Kaul et al., 2016).

Thus, not surprisingly, a substantial amount of work has been done in the past to characterize the structure and function of root endophytes (Bulgarelli et al., 2012, 2015; Lundberg et al., 2012; Peiffer et al., 2013; Edwards et al., 2015). However, most current studies focused on the presence of resident communities. Yet it is important to note that some “opportunistic endophytes” (Hardoim et al., 2008) or “passenger endophytes” (de Almeida et al., 2009) may enter the plant endosphere just by chance. Their functional contributions to the community could be limited, if they are only transient or dormant. A previous study has shown that the active endophytic groups were less complex than the resident community (Reiter et al., 2003). Therefore, more study of the active endophytic community is needed toward a better understanding of plant and endophyte interaction.

It was demonstrated that seed-borne endophytes are able to persist in the seedlings as almost all genera isolated from seeds were also recovered from bean roots (Lopez-Lopez et al., 2010). Hardoim et al. (2012) showed that seed endophytes of rice are important founders of bacteria colonizing the root interior using a fingerprinting method. Bacteria from the external environment, basically soil, will also colonize plants, leading to shifts in bacterial community structure during root development (Kristin and Miranda, 2013). However, the dynamics of seed-borne endophytes during seed germination and root development are still not clear.

A recent study indicated that seed associated microorganisms may release seed dormancy through production of cytokinins (Goggin et al., 2015). Puente et al. (2009) demonstrated that bacterial endophytes from cactus seeds could improve the establishment of seedlings on barren rocks. Seedling development was stopped when disinfecting cactus seeds with antibiotics. However, although the seed associated microbiome obviously strongly impacts plant growth and health, little is known about the structure and regulators of seed associated microbiome.

In this study, we focused on the potentially active bacterial community. We investigated (a) plant cultivar dependent effects of the seed microbiome (b) the role of the seed microbiome as “first inoculum” of root endophytes and (c) the stability of this “first inoculum” during plant development. We used different cultivars of barley as a model and performed a greenhouse experiment using soil as well as experiments in axenic systems using sterile sand mixture. Bacterial communities were analyzed from surface sterilized seeds and roots using barcode sequencing based on rRNA. We postulate (a) cultivar dependent differences in the seed microbiome structure are low and (b) that the seed microbiome will make a significant part of the root microbiome at early plant growth stages, being further substituted by bacterial populations present in the rhizosphere.

## Materials and Methods

### Seeds Surface Sterilization

In the frame of this study, we used barley cultivars Alexis, Barke, Marthe, Salome, and Simba. Alexis and Barke were obtained from Saatzucht Breun GmbH & Co. KG (Herzogenaurach, Germany), while Marthe, Salome, and Simba were supplied by Nordsaat Saatzucht GmbH (Langenstein, Germany). Surface sterilization of seeds was performed using 70% ethanol for 5 min and 2% NaClO for 20 min. This method was selected because a microscopic comparison showed that this method is more efficient in removing surface microbes than commonly used ultrasonication and shaking (Reinhold-Hurek et al., 2015). Detailed procedures of the surface sterilization have been described previously (Kutter et al., 2006). The success of the surface sterility for seeds was checked by FISH using Eub-335-I, Eub-335-II, and Eub-338-III (Metabion, Germany) as described elsewhere (Spohn et al., 2015) and plating on R2A agar plates.

### RNA and DNA Co-extraction from Seeds

After plating on R2A agar plates for 24 h at 23°C in dark, the imbibed seeds were used for nucleic acid extraction. Each sample was composed of six seeds, which were grounded using liquid nitrogen with a mortar and pestle. 0.1 g from the seed powder was used for a coextraction of DNA and RNA using Griffiths’ protocol (Griffiths et al., 2000). Extraction was performed for each cultivar in five replicates (each consisting of six seeds). Water served as a negative control and was used for extraction of nucleic acids in a parallel approach.

DNA/RNA co-extracts were digested with DNase Max^TM^ Kit (MoBio, United States) to obtain pure RNA. Complete DNA digestion was checked and confirmed with real time quantitative PCR for 16S rRNA genes using the primer set 968F/1401R. The resulting purified RNA was reverse transcribed into cDNA using the High-Capacity cDNA Reverse Transcription Kit (Applied Biosystems, United States). The other aliquot was left untreated and is from here on referred to as DNA. DNA and cDNA samples were stored at -80°C until further analysis.

### Barley Cultivation

For barley cultivation, surface sterilized seeds were germinated on a wet paper in Petri dishes in the dark for 3 days at 30°C. In this work, we used two systems to investigate the impact of seed-borne endophytes on the composition of the root associated microbiome. To study the root endophytes originating from seeds, we created axenic systems where there are no external microbes. We also used soil based systems, which resemble natural conditions, to investigate to what extent seed-borne endophytes can persist in roots when microbes from the rhizosphere also colonize the root interior.

Axenic systems were made using sterile beakers (250 ml), sterile glass beads (185 g) and 45 ml sterile MS media (Duchefa Biochemie bv, The Netherlands). Six germinated seeds were put in the glass beads and covered with another sterile beaker. The complete system was then sealed with Parafilm. Five replicates (each consisting of six seeds) were used per cultivar. Plants were grown in a climate chamber under controlled conditions (23°C/14 h, 15°C/10 h, and 65% humidity).

For “soil based systems,” germinated seeds were sown in pots filled with sandy soil. The soil was collected from the top layer from an arable field in Scheyern Research Farm (Scheyern, Germany) in July, 2014 and was sieved using a 2 mm mesh. The pots were 13 cm high, with the top square 13 × 13 cm and 9.6 × 9.6 cm at the bottom. The soil was filled to a depth of 10 cm in the pot. Every pot contained one well-germinated seed. For each cultivar four replicates were prepared. The plants were grown in a greenhouse under controlled conditions with 12 h light at 20°C and 12 h dark at 16°C. The plants were watered twice a week to obtain a water content of 60% of the maximal water holding capacity.

### Roots Sampling and Surface Sterilization

We used Zadoks decimal code (Zadoks et al., 1974) for the growth stages scale and determined our sampling time accordingly. Barley plants growing in axenic systems were sampled 8 days (seedling growth, Z13) after sowing the seeds. Plants growing in the greenhouse were harvested at two time points, 2 weeks after planting (seedling growth, Z13) and 10 weeks after planting (booting, Z41). Before surface sterilization the remaining sand/soil from the roots was removed by shaking and washing in water.

Roots were sterilized like described above for seeds, washed five times with sterile water and shock frozen using liquid nitrogen. Root samples were grounded to powder using the TissueLyzer II (Qiagen, Germany) according to the manufacturer’s instructions. RNA extraction, reverse transcription and sample handling was done as described above.

### Library Preparation and Sequencing

In the frame of this project, primer pair S-D-Bact-0008-a-S-16 (Muyzer et al., 1993) and S-D-Bact-0343-a-A-15 (Alm et al., 1996) was used (Klindworth et al., 2013). As preliminary data indicated a huge co-amplification of plastids when DNA was used as a target, we used RNA in this study, as plastid content in rRNA is low (Supplementary Figure S1). To compare the resident and active community, we also performed DNA amplification using the primer 338F/789R, which was reported to exclude chloroplast amplification (Dorn-In et al., 2015). Our data indicated a higher number of genera in 16S rRNA sequences when primer pair S-D-Bact-0008-a-S-16 and S-D-Bact-0343-a-A-15 was used to amplify the obtained cDNA compared to DNA amplification using 338F/789R. It also confirmed a strong bias of the primer 338F/789R, which was mainly a result of the predominance of Enterobacteriaceae, whereas the percentage of Enterobacteriaceae was much lower in the rRNA samples (Supplementary Figure S2). Therefore, we chose the primer pair S-D-Bact-0008-a-S-16 and S-D-Bact-0343-a-A-15 for the analysis of the active fraction of the community.

The PCR conditions were the following: 98°C for 5 min, followed by 30 cycles each at 98°C for 10 s, 60°C for 30 s and 72°C for 30 s, followed by 72°C for 5 min. Triplicate amplicons were pooled and purified using Agencourt AMPure XP kit (Beckman Coulter, United States). DNA quantity was assessed with the Quant-iT PicoGreen dsDNA Assay Kit (Invitrogen, United States). Nextera XT Index Kit v2 (Illumina, United States) was used for amplicon indexing. Reactions were kept at 98°C for 5 min, followed by eight cycles at 98°C for 10 s, 55°C for 30 s and 72°C for 30 s, with a final extension step of 10 min at 72°C. All amplicons were purified and quantified as described above. The purified amplicons were then pooled in 4 nM concentrations and sequenced on Illumina Miseq platform (Illumina, United States). The obtained sequences were deposited under the accession number SRP102191 in the SRA.

### Data Analysis

The sequencing analysis was performed with the software QIIME (version 1.9.0) (Caporaso et al., 2010). Adaptors and primers were removed using AdapterRemoval (Lindgreen, 2012). Phix contamination was removed using the program Deconseq (Schmieder and Edwards, 2011). Reads were merged and filtered by size (according to primer set) and quality (Phred quality score > 2). The sequences were then clustered into operational taxonomic units (OTUs) using an open reference strategy based on 97% identity with GreenGenes Database (13_5 release) (DeSantis et al., 2006) as reference. Taxonomy was assigned with RDP classifier (Wang et al., 2007) retrained with GreenGenes 16S rRNA database (13_5 release). OTUs assigned to chloroplast were filtered out.

The statistical analysis was also performed using QIIME (version 1.9.0). Plots were generated with R (version 3.2.1) using packages vegan, plyr, beanplot, ggplot, and vcd.

## Results

### Sequencing Summary

A total of 7,838,588 raw sequences were obtained. The number of reads per sample ranged from 11,901 to 199,129. After adaptor, primer and chimera removal as well as length and quality filtering, 6,547,064 high-quality reads were clustered at 97% sequence identity. OTUs assigned to chloroplast were discarded, resulting in 5,816,127 remaining reads. Low abundant OTUs (less than 0.005%) were filtered out, resulting in 851 OTUs. To compare the diversity in different samples, we rarefied the data to 11,390 reads per sample for comparison. Rarefaction curves indicated that the sequencing depth is sufficient to capture the microbial diversity (Supplementary Figure S3).

### Active Bacterial Groups in Seeds

**FIGURE 1:**
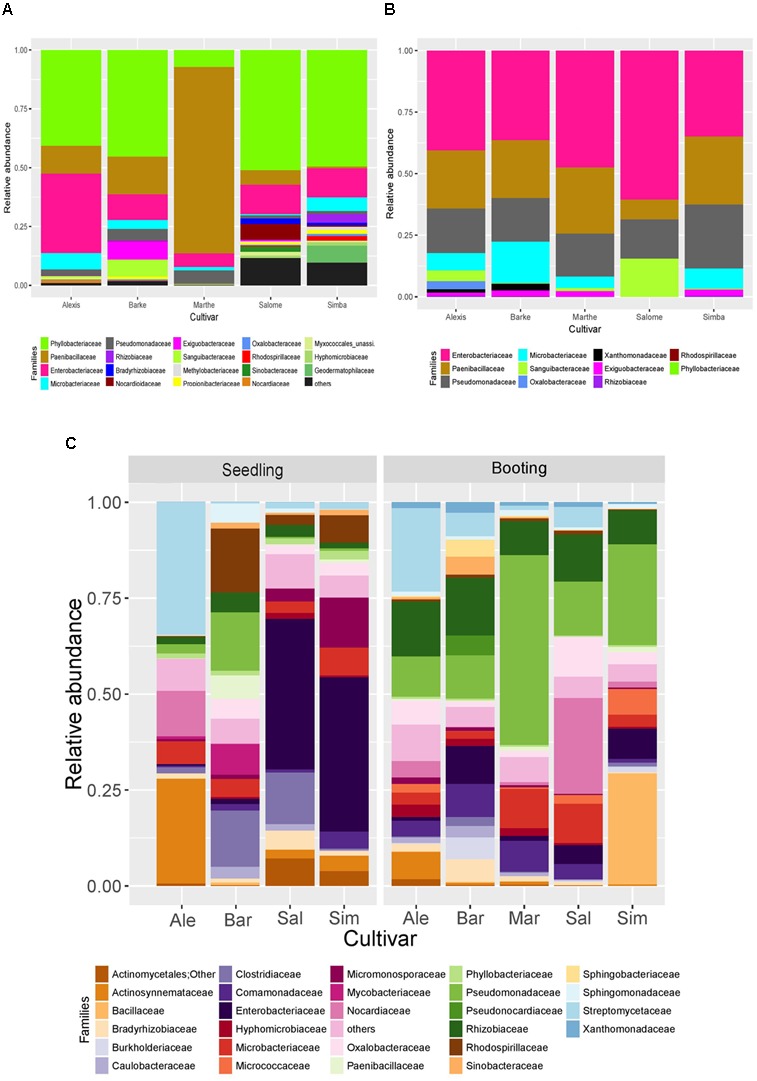
The structure of bacterial communities associated with **(A)** seeds **(B)** roots growing in axenic systems and **(C)** roots growing in greenhouse at family level. Ale, Alexis; Bar, Barke; Sal, Salome; Mar, Marthe; Sim, Simba (*n* = 3–5). OTUs with abundance less than 0.005% were filtered out.

**FIGURE 2:**
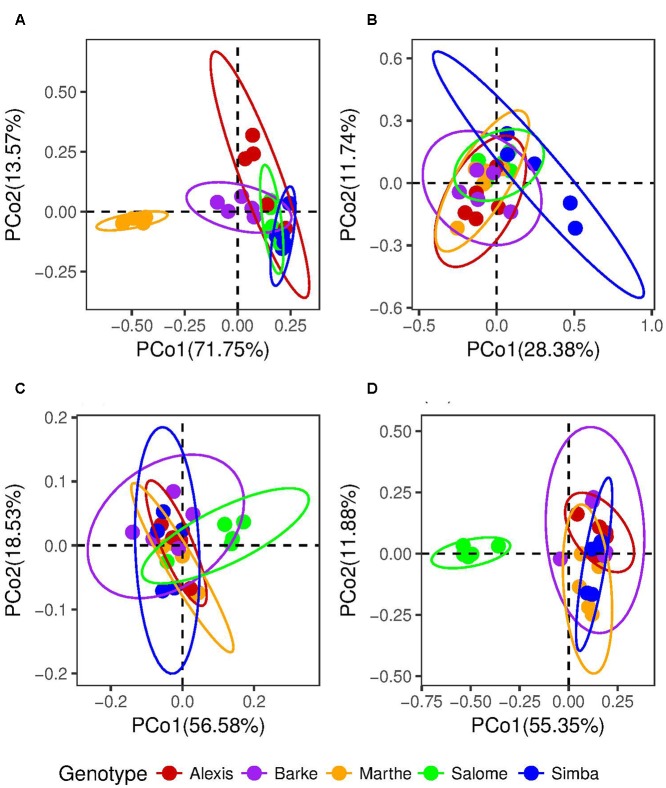
Principal coordinate analysis (PCoA) plot to investigate the differnce of microbiome associated with seeds and roots (*n* = 3–5). **(A)** Seed samples based on weighted Unifrac and **(B)** unweighted Unifrac distance metrics; **(C)** root samples from axenic systems based on weighted Unifrac and **(D)** unweighted Unifrac distance metrics. The lines of ellipses show 95% confidence intervals.

For the active seed associated microbiome, we identified 137 genera from 83 families of 10 different phyla based on our molecular barcoding approach (**Figure 1A**). To investigate the genotype effect on the active seed associated microbiome, we carried out principal coordinate analysis (PCoA) both based on weighted and unweighted Unifrac distance metrics (**Figures 2A,B**). Permutational multivariate analysis of variance using distance matrices (ADONIS) showed significant differences between active bacterial communities across cultivars (weighted Unifrac, *p* = 0.001, *R*^2^ = 0.81; unweighted Unifrac, *p* = 0.001, *R*^2^ = 0.31). We found two OTUs which differed in frequencies across all cultivars using Kruskal–Wallis test (Bonferroni corrected *p*-value < 0.05). These two OTUs were assigned to *Paenibacillus* and *Pseudomonas*.

Despite of these differences, we observed a shared set of associated bacteria. 21 core OTUs were found in all cultivars, which were assigned to Phyllobacteriaceae (four OTUs), Paenibacillaceae (five OTUs), Enterobacteriaceae (five OTUs), Pseudomonadaceae (three OTUs), Oxalobacteraceae (one OTU), Comamonadaceae (one OTU), Xanthomonadaceae (one OTU), and Propionibacteriaceae (one OTU) (**Table 1**). These core OTUs represented, in total, more than 50% of all reads. Notably, five OTUs assigned to Phyllobacteriaceae, Paenibacillaceae, Pseudomonadaceae, and Enterobacteriaceae, respectively contributed to most of the reads, while others had relative abundances of less than 1%.

**Table 1:** The relative abundance and taxonomy assignment of core OTUs in seed associated microbiome.

OTU ID	Relative abundance in each genotype (%)					Taxonomy				
	Alexis	Barke	Marthe	Salome	Simba	Phylum	Class	Order	Family	Genus
OTU219107	34.77	42.63	6.91	54.97	50.67	Proteobacteria	α-Proteobacteria	Rhizobiales	Phyllobacteriaceae	*Phyllobacterium*
OTU1095	0.74	0.99	0.21	1.22	1.20	Proteobacteria	α-Proteobacteria	Rhizobiales	Phyllobacteriaceae	*Phyllobacterium*
OTU431921	0.14	0.17	0.03	0.26	0.24	Proteobacteria	α-Proteobacteria	Rhizobiales	Phyllobacteriaceae	*Phyllobacterium*
OTU705063	0.03	0.06	0.01	0.55	0.05	Proteobacteria	α-Proteobacteria	Rhizobiales	Phyllobacteriaceae	*Phyllobacterium*
OTU101	9.21	10.76	59.86	3.08	0.68	Firmicutes	Bacilli	Bacillales	Paenibacillaceae	*Paenibacillus*
OTU415	1.44	2.74	7.53	0.66	0.13	Firmicutes	Bacilli	Bacillales	Paenibacillaceae	*Paenibacillus*
OTU537	0.36	0.65	4.05	0.94	0.02	Firmicutes	Bacilli	Bacillales	Paenibacillaceae	*Paenibacillus*
OTU863	0.32	2.12	3.21	0.28	0.02	Firmicutes	Bacilli	Bacillales	Paenibacillaceae	*Paenibacillus*
OTU1508	1.59	0.05	0.01	0.69	0.06	Firmicutes	Bacilli	Bacillales	Paenibacillaceae	*Saccharibacillus*
OTU725048	1.68	3.13	2.26	1.86	1.71	Proteobacteria	γ-Proteobacteria	Enterobacteriales	Enterobacteriaceae	*Trabulsiella*
OTU1109844	0.02	0.04	0.01	0.07	0.05	Proteobacteria	γ-Proteobacteria	Enterobacteriales	Enterobacteriaceae	*Trabulsiella*
OTU667	0.75	1.82	0.94	3.83	0.04	Proteobacteria	γ-Proteobacteria	Enterobacteriales	Enterobacteriaceae	*Erwinia*
OTU289261	0.76	1.21	0.21	1.58	1.35	Proteobacteria	γ-Proteobacteria	Enterobacteriales	Enterobacteriaceae	NA (not assigned)
OTU541119	0.16	0.20	0.05	0.20	0.15	Proteobacteria	γ-Proteobacteria	Enterobacteriales	Enterobacteriaceae	NA (not assigned)
OTU578606	0.36	2.36	4.96	0.67	0.47	Proteobacteria	γ-Proteobacteria	Pseudomonadales	Pseudomonadaceae	*Pseudomonas*
OTU791973	0.88	0.45	0.03	0.23	0.12	Proteobacteria	γ-Proteobacteria	Pseudomonadales	Pseudomonadaceae	*Pseudomonas*
OTU541859	0.06	0.13	0.45	0.09	0.04	Proteobacteria	γ-Proteobacteria	Pseudomonadales	Pseudomonadaceae	*Pseudomonas*
OTU299883	0.23	0.34	0.04	0.38	0.14	Proteobacteria	β-Proteobacteria	Burkholderiales	Oxalobacteraceae	*Ralstonia*
OTU264546	0.12	0.15	0.01	0.10	0.11	Proteobacteria	β-Proteobacteria	Burkholderiales	Comamonadaceae	*Delftia*
OTU345540	0.09	0.17	0.02	0.14	0.08	Proteobacteria	γ-Proteobacteria	Xanthomonadales	Xanthomonadaceae	*Stenotrophomonas*
OTU165421	0.47	0.97	0.19	0.89	1.48	Actinobacteria	Actinobacteria	Actinomycetales	Propionibacteriaceae	*Propionibacterium*

### Active Bacterial Groups in Roots

The active bacteria associated with roots growing in axenic systems differed significantly in α diversity across cultivars (*p* < 0.05) (Supplementary Figure S4). Differences in ß diversity were also detected in both weighted (ADONIS, *p* = 0.002, *R*^2^ = 0.43) and unweighted (ADONIS, *p* = 0.001, *R*^2^ = 0.66) Unifrac distance metrics (**Figures 2C,D**). Only five core OTUs were found, which were assigned to Enterobacteriaceae and Pseudomonadaceae (**Table 2** and **Figure 1B**). Interestingly, these families are also the most abundant families in the seed associated microbiome.

**Table 2:** The relative abundance and taxonomy assignment of core OTUs in microbiome associated with seedlings growing in axenic systems.

OTU ID	Relative abundance in each genotype (%)					Taxonomy				
	Alexis	Barke	Marthe	Salome	Simba	Phylum	Class	Order	Family	Genus
OTU 791973	11.91	10.88	10.97	0.004	19.39	Proteobacteria	Gammaproteobacteria	Pseudomonadales	Pseudomonadaceae	*Pseudomonas*
OTU 578606	0.33	1.69	1.76	8.78	3.16	Proteobacteria	Gammaproteobacteria	Pseudomonadales	Pseudomonadaceae	*Pseudomonas*
OTU 541859	0.06	0.13	0.64	5.84	0.16	Proteobacteria	Gammaproteobacteria	Pseudomonadales	Pseudomonadaceae	*Pseudomonas*
OTU 725048	29.89	25.06	36.50	0.02	25.85	Proteobacteria	Gammaproteobacteria	Enterobacteriales	Enterobacteriaceae	NA (not assigned)
OTU 554163	0.12	0.09	0.17	0.29	0.07	Proteobacteria	Gammaproteobacteria	Enterobacteriales	Enterobacteriaceae	NA (not assigned)

**FIGURE 3:**
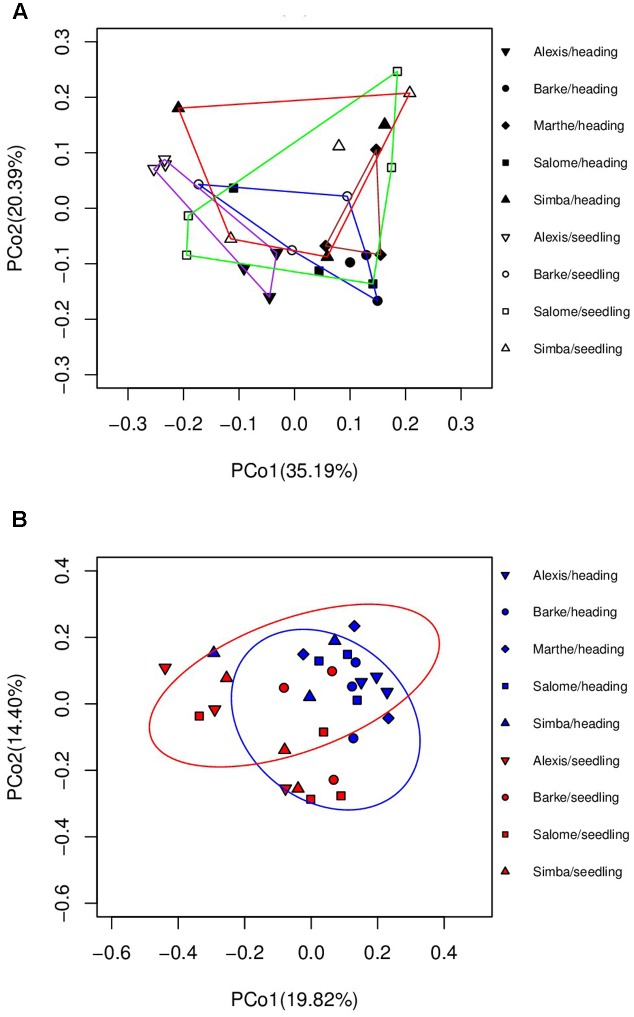
Principal coordinate analysis plot to investigate the differnce of root microbiome in greenhouse based on **(A)** weighted Unifrac and **(B)** unweighted Unifrac distance metrics.

We further analyzed the active groups associated with roots growing in the soil (**Figure 1C**). We also investigated the influences of genotype and growth stage. Statistical analysis (ADONIS) using weighted Unifrac distances, revealed both genotype and growth stage dependent impacts on barley endophytes (genotype, *p* < 0.05, *R*^2^ = 0.23; growth stage, *p* < 0.05, *R*^2^ = 0.10) (**Figure 3A**).

However, when unweighted Unifrac metrics were used, the genotype effects were not significant (*p* > 0.05). Only the growth stage accounted for the variation between microbial communities significantly (*p* = 0.001, *R*^2^ = 0.124). Consistently, clustering patterns were observed only by growth stages in the ordination plot of PCoA (**Figure 3B**), implying that the plants’ developmental stage is the main driving factor in shaping the root associated bacterial community.

To gain insights into the richness of barley root microbiota, we compared the number of observed OTUs and the Chao1 index of the community retrieved from seedling (2 weeks) and booting stage (10 weeks) (Supplementary Figure S5). Endophytes at the booting stage were significantly more diverse, resulting in a higher Chao1 index (*t*-test, *p* = 0.002).

We found 16 core OTUs at seedling stage and 67 at booting stage (Datasheet S1). Although there was a large overlap between the OTUs at two growth stages, only 10 OTUs were common across all cultivars and growth stages in the root associated microbiome, which were assigned to Bradyrhizobiaceae (two OTUs), Comamonadaceae (two OTUs), Phyllobacteriaceae (two OTUs), Actinosynnemataceae (one OTU), Propionibacteriaceae (one OTU), Caulobacteraceae (one OTU), and Rhizobiaceae (one OTU) (**Table 3**). In general, these OTUs were not abundant, most of which had less than 1% of the total reads.

**Table 3:** The relative abundance and taxonomy assignment of core OTUs shared across all cultivars and growth stages in microbiome associated with roots growing in greenhouse.

OTU ID	Relative abundance (%)									Taxonomy				
	2 weeks (seedling statage, Z13)				10 weeks (booting stag, Z41)						Phylum	Class	Order	Family	Genus
	Ale	Bar	Sal	Sim	Ale	Bar	Mar	Sal	Sim
OTU15	21.23	0.10	1.85	4.12	0.04	0.34	0.62	0.02	0.35	Actinobacteria	Actinobacteria	Actinomycetales	Actinosynnemataceae	NA (not assigned)
OTU165421	0.03	0.43	0.18	0.16	0.14	0.16	0.06	0.04	0.10	Actinobacteria	Actinobacteria	Actinomycetales	Propionibacteriaceae	*Propionibacterium*
OTU303643	0.01	0.82	0.25	0.04	0.19	0.21	0.14	0.10	0.08	Proteobacteria	α-Proteobacteria	Caulobacterales	Caulobacteraceae	NA (not assigned)
OTU826270	0.73	0.54	5.38	0.88	0.92	0.65	0.45	0.12	0.15	Proteobacteria	α-Proteobacteria	Rhizobiales	Bradyrhizobiaceae	*Bradyrhizobium*
OTU523224	0.05	0.05	0.16	0.09	0.17	0.23	0.10	0.07	0.02	Proteobacteria	α-Proteobacteria	Rhizobiales	Bradyrhizobiaceae	NA (not assigned)
OTU102142	0.13	0.11	0.11	0.01	0.41	0.16	0.82	1.98	1.02	Proteobacteria	α-Proteobacteria	Rhizobiales	Rhizobiaceae	NA (not assigned)
OTU705063	0.07	0.07	0.42	0.06	0.15	0.15	0.09	0.22	0.28	Proteobacteria	α-Proteobacteria	Rhizobiales	Phyllobacteriaceae	*Mesorhizobium*
OTU806201	0.02	0.06	1.09	0.18	0.34	0.21	0.20	0.07	0.04	Proteobacteria	α-Proteobacteria	Rhizobiales	Phyllobacteriaceae	*Mesorhizobium*
OTU819037	0.04	0.11	0.04	0.06	0.33	0.67	4.10	1.89	0.18	Proteobacteria	β-Proteobacteria	Burkholderiales	Comamonadaceae	*Variovorax*
OTU590047	0.01	0.84	0.07	0.03	1.90	0.48	0.84	0.36	0.20	Proteobacteria	β-Proteobacteria	Burkholderiales	Comamonadaceae	NA (not assigned)

### Comparing Seed Microbiome and Root Microbiome

In axenic systems, 18 OTUs were only detected in the roots from plants but not in the seeds. However, at genus level, one genus detected in the roots (*Xanthomonas*) was not found in the seeds. A genotype effect was observed for both seed and root associated microbiomes. This pattern changed when the seeds developed into plants in axenic systems. Marthe had the most divergent seed microbiome while Salome showed the biggest difference in the composition of the root associated communities compared to the other cultivars (**Figures 2A,D**).

**FIGURE 4:**
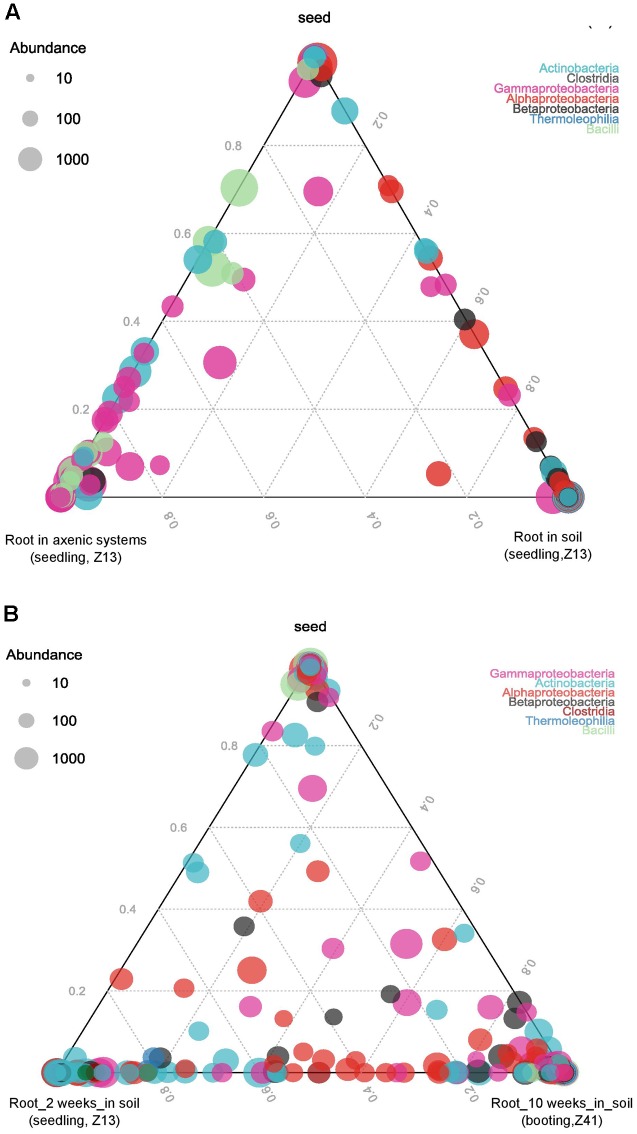
Ternary plot of OTUs showing the distribution of OTUs in different sample categories. **(A)** Seeds and root seedlings (Z13) grown in axenic systems and soil. **(B)** Seeds and roots grown in soil at seedling (Z13) and booting (Z41) stage. Each corner of the triangle represents a sort of sample. The size of plotted dots corresponds to the abundance of the OTUs. The dashed grid lines inside the plot indicate the contribution of each sample type (*n* = 3–5).

All root endophytes shared OTUs with the seed associated microbiome, regardless of their growing conditions. However, plants growing in soil shared fewer OTUs with seeds compared to plants growing in axenic systems (**Figure 4A**), indicating a strong influence of soil microbiota. For plants grown in soil, root endophytes shared OTUs with the seed microbiome, in both seedling and booting stage. But more OTUs were found to overlap between the two growth stages than between the root and seed microbiome (**Figure 4B**).

**FIGURE 5:**
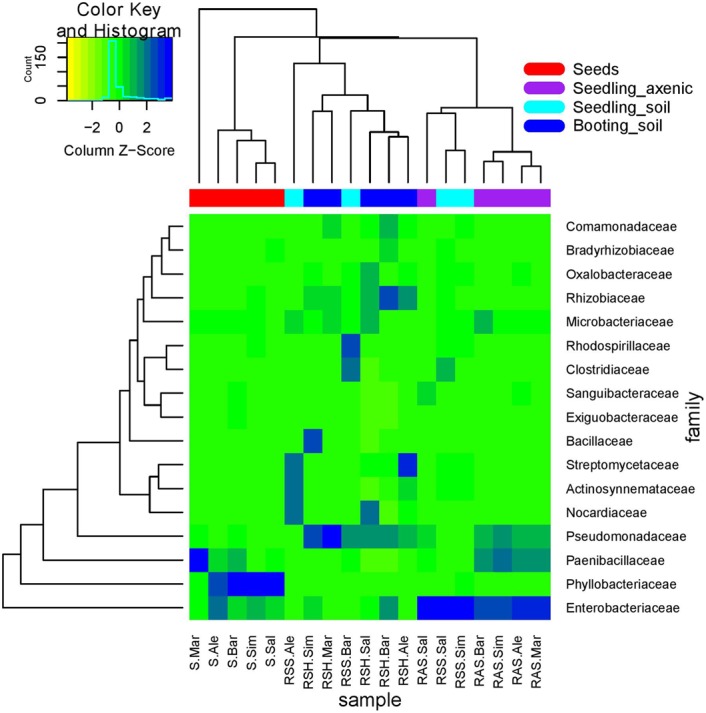
Heat map of the relative abundance of the most abundant families present in seed and root associated microbiome. S, seed; RAS, roots growing in axenic systems (seedling, Z13); RSS, roots growing in soil (seedling, Z13); RSH, roots growing in soil (booting, Z41). Ale, Alexis; Bar, Barke; Sal, Salome; Mar, Marthe; Sim, Simba.

The heatmap further illustrates the dynamics of bacterial communities (**Figure 5**). Enterobacteriaceae were abundant in both seed microbiome and root microbiome in axenic systems. In plants grown in soil, the abundance of Enterobacteriaceae varied across cultivars. Barke and Simba showed a higher abundance of Enterobacteriaceae at the seedling stage (Z13) while Salome and Simba showed a higher abundance at the booting stage (Z41). Phyllobacteriaceae, which was the most abundant family in the seed microbiome, decreased dramatically to less than 2% in the root associated microbiome. In contrast, Pseudomonadaceae were largely enriched in the root associated microbiota. Similarly, an enrichment of Rhizobiaceae was also observed in the root microbiome, but only when plants were grown in soil. Streptomycetaceae, detected in low abundance in seeds and not found in roots growing in axenic systems, appeared to be abundant in roots growing in soil. On the contrary, Paenibacillaceae, highly abundant in both seeds and roots growing in axenic systems, decreased to negligible percentage in roots growing in soil.

## Discussion

### Seed Associated Microbiome

In this work, we investigated modern commercially available barley cultivars. A significant cultivar effect was observed in the seed associated microbiome. This result was unexpected as we assumed that bacteria colonizing the seed interior are subjected to similar selective pressure and, hence, would not significantly differ between the cultivars. In fact, many studies have shown that the plant cultivar is less relevant for the composition of bacterial communities, whereas the plant compartment plays a major role (Bulgarelli et al., 2012). However, they only analyzed the resident bacteria, while our work studied the potentially active part of the community. The influence of the plant genotype is probably stronger on the potentially active endophytes than on the total community. We also consider that the differences observed in our analysis were driven by the extremely high abundance of *Paenibacillus* sequences in the libraries obtained from Marthe, which were not found in other cultivars.

We observed a dominance of a few bacterial OTUs assigned to *Phyllobacterium* (OTU219107), *Paenibacillus* (OTU101), and *Trabusiella* (OTU725048) in the seeds of the five investigated cultivars. *Phyllobacterium* has been described as a plant-associated genus and was isolated from the rhizosphere, root and nodules from different plant species (Mantelin et al., 2006). It was also shown to be vertically transmitted in *Phaseolus vulgaris* (Lopez-Lopez et al., 2010). Although their role in seeds was not yet investigated, *Phyllobacterium* was shown to promote root growth in *Brassica napus* and *Arabidopsis thaliana* (Bertrand et al., 2001; Contesto et al., 2010; Kechid et al., 2013).

Some *Paenibacillus* strains produce cytokinins (Timmusk and Wagner, 1999), which are directly involved in seed germination (Kumar et al., 2014). Goggin et al. (2015) showed that the reduction of the density of endophytic populations, e.g., by heating, made seeds unable to lose dormancy. They postulated that this was caused by a decrease in the concentration of cytokinins of bacterial origin. In fact, the inoculation of *A. thaliana* with a *Paenibacillus polymyxa* strain reduced the germination time (Kefela et al., 2015).

Moreover, bacteria were shown to alleviate reactive oxygen species (ROS) stress, allowing quinoa seeds to germinate even under hostile environmental conditions (Pitzschke, 2016). It is known that ROS, namely hydrogen peroxide, induces a mitogen-activated protein kinases (MAPKs) dependent decrease of abscisic acid content, a hormone known to inhibit germination (Barba-Espin et al., 2011). H_2_O_2_ also acts as a priming factor that promotes changes on seed proteome, which may relieve seeds from dormancy (Oracz et al., 2008). Nevertheless, at higher concentrations, ROS may cause tissue damage. Therefore, for germination to occur, it is necessary that ROS are kept at a certain level, the so-called “oxidative window.” Although not shown for seeds, bacteria from the genus *Paenibacillus* were shown to reduce oxidative stress in legume nodules (Rodrigues et al., 2013).

*Trabusiella* was also shown to contribute with a great part to the seed microbiome in barley. The two species described within the genus *Trabusiella* are not plant associated bacteria. However, *Trabusiella* OTUs and other genera within the family Enterobacteriaceae were also found in high abundance in seeds from Agave and many other plant species (Truyens et al., 2015; Coleman-Derr et al., 2016). It was postulated that seed associated Enterobacteriaceae reduce the concentration of seed exudates that trigger the sporulation of fungal pathogens, such as *Phytum ultimatum* (Hood et al., 1998). Proteome analyses showed that during germination barley seeds synthetize and secrete a range of protease inhibitors, probably for the neutralization of fungal exoenzymes (Sultan et al., 2016). Vertical transmission of bacteria that reduce the pathogen sporulation may be another mechanism by which barley plants control infection.

### Root Associated Microbiome

In this work, we used two systems to grow barley: axenic systems with sterile sand mixture and greenhouse systems with natural soil.

We observed significant differences on the composition of the microbiome detected in roots of the five cultivars growing in axenic systems. Compared to seeds, we noted a shift in the taxonomical composition. *Phyllobacterium*, *Paenibacillus*, both highly abundant in the seeds, were less numerous in the axenic roots. On the other hand, bacteria belonging to the genera *Pseudomonas* and *Trabusiella* were found largely enriched in root tissue. Two major OTUs, OTU 791973 (*Pseudomonas*) and OTU 725048 (*Trabusiella*), were found in all root and seeds samples. Many strains of these two families were reported to promote plant growth, and were frequently described to be found in roots as well (Bulgarelli et al., 2013; Cope-Selby et al., 2016).

In contrast, cultivar dependent effects were less pronounced in roots growing in soil, and were only significant when calculating the distance between samples using weighted Unifrac metrics. Our results indicate that the divergence of root microbiota across genotypes is only quantitative. The variation between the genotypes was manifested in the abundance of many OTUs from diverse taxa (Streptomycetaceae, Comamonadaceae, Rhizobiaceae, and Nocardiaceae), rather than by the presence/absence of single OTUs in the given genotypes.

These findings are in accordance with a recent study comparing the resident root microbiota of wild and domesticated barley, where a small but significant host genotype effect on the basis of abundance was reported (Bulgarelli et al., 2015). We suppose that the genetic variation across our genotypes is smaller than that in the above study of Bulgarelli et al. (2015) which compared wild and domesticated barley. Therefore, less variation of the associated microbiome is expected. Yet we still observed a significant impact of the plant cultivar, though only quantitatively, indicating that host genotype is an important filter for the active communities inside plants.

Interestingly, OTUs found in the roots of all plants grown in arable soil were in low abundance and differed from those detected in the axenic systems. The different cultivars grown in the same soil were colonized by bacteria belonging to same taxa, but not exactly the same OTUs. This might be a reflection of the great diversity and functional redundancy found in soils. Furthermore, we observed an enrichment of Actinobacteria in roots of plants grown in soil. Actinobacteria are known to produce a number of secondary metabolites that may hamper the growth of other bacteria, including plant pathogens (Palaniyandi et al., 2013). They were also shown to be enriched in the endophytic compartments of *A. thaliana* (Lundberg et al., 2012). Nevertheless, members of the family Pseudomonadaceae were the only bacteria found in high abundance in root tissue independent from growth condition or plant development stage, suggesting a sturdy association of *Pseudomonas* sp. with barley roots.

## Conclusion

In this study, we characterized active bacterial communities associated with seeds and roots from five commercially available barley cultivars. We found that the genotype is a significant driving factor in shaping the seed associated microbiome. When plants were grown in soil, the developmental stage was found to have a more pronounced impact on the active community composition, whereas the genotype effect was only quantitative. A conserved set of core OTUs was identified, which comprises stable community members belonging to 12 families including Phyllobacteriaceae, Enterobacteriaceae, Pseudomonadaceae, and Propionibacteriaceae. Seed endophytes were an important inoculum for bacterial communities in the roots in early growth stages. Yet, we observed a large shift when the roots develop from seedling to booting stage in soil. Two OTUs assigned to *Phyllobacterium* were found in all seeds and root samples growing in soil, indicating a relationship between seed-borne and root associated microbiome in barley.

Thus, future studies should be more related to the functions of the seed and root associated microbiome, to clarify their role for plant development and health. Other parts of the microbiome, e.g., fungi, should also be assessed in the future to get an overall overview on the plant associated microbiome.

## Author Contributions

LY: this author contributed with the experimental design, laboratory work, data analyses, result discussion and text writing. JD: this author participated of the experimental design, laboratory work and data analyses. AS: this author participated of the data analyses and manuscript writing. PS: this author contributed with data analyses, results discussion and manuscript writing. MS: this author contributed with data analyses, results discussion and manuscript writing. VR: this author contributed with the experimental design, data analyses, results discussion and text writing.

## Conflict of Interest Statement

The authors declare that the research was conducted in the absence of any commercial or financial relationships that could be construed as a potential conflict of interest.
