# Supplementary material for: Dominant Groups of Potentially Active Bacteria Shared by Barley Seeds become Less Abundant in Root Associated Microbiome
Source: Front Plant Sci. 2017 Jun 15;8:1005. doi: 10.3389/fpls.2017.01005 (PMC5471333; doi:10.3389/fpls.2017.01005)
Supplement: Supplementary file 1 [file Data_Sheet_1.DOCX]

**Figure legends**

Figure S1 16S rRNA amplicon sequencing based on cDNA or DNA with universal primer.

Figure S2 Taxonomic composition comparison of DNA and rRNA based method in root and seed associated microbiome.

Figure S3 Rarefaction curve at subsampling depth of 11390 reads per sample

Figure S4 α- diversity of the microbiome associated with roots in axenic systems (n=5).

Figure S5 Bacterial richness detected for in the roots of barley plants grown in soil during seedling (Z13, 2 weeks) and booting (Z41, 10 weeks).

Figure S1 16S rRNA amplicon sequencing based on cDNA or DNA with universal primer.


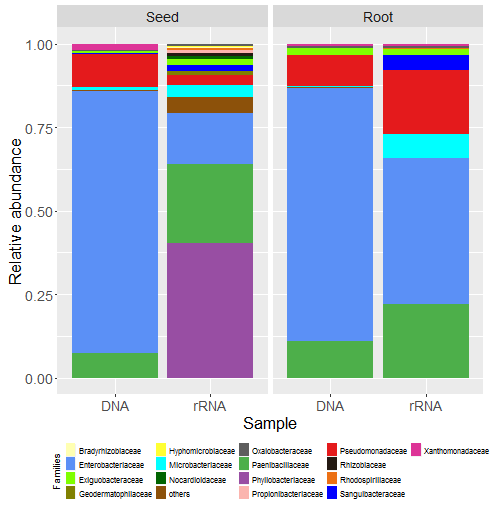


Figure S2 Taxonomic composition comparison of DNA and rRNA based method in root and seed associated microbiome.


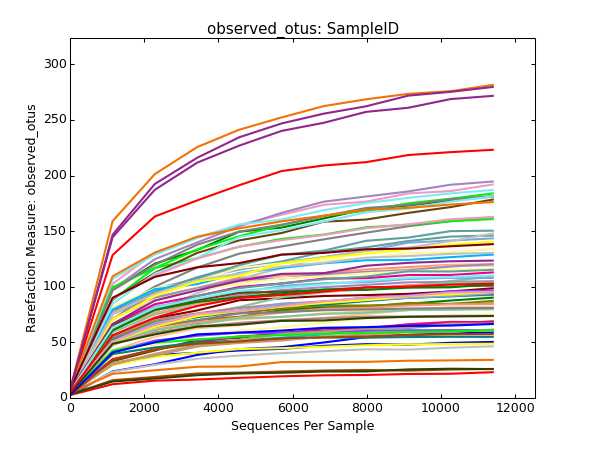


Figure S3 Rarefaction curve at subsampling depth of 11390 reads per sample


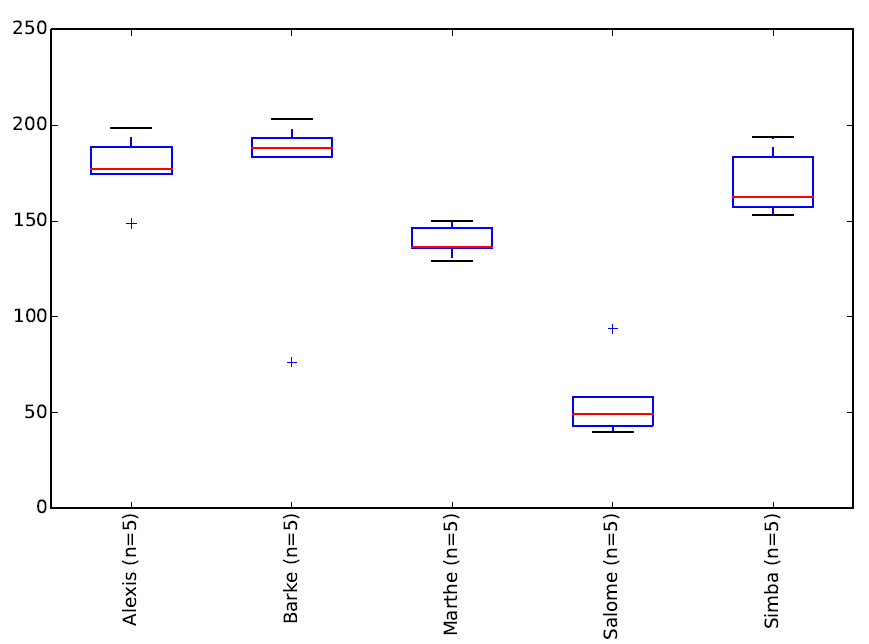


Figure S4 α- diversity of the microbiome associated with roots in axenic systems (n=5).


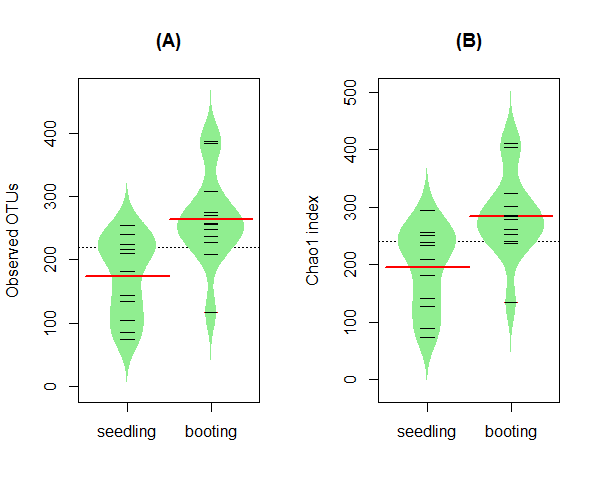


Figure S5 Bacterial richness detected for in the roots of barley plants grown in soil during seedling (Z13, 2 weeks) and booting (Z41, 10 weeks). Observed OTUs (A) and higher Chao1 index (B). Each sample is represented by a black line. while the green area represents the estimation of the distribution. Red line indicates the average level (n=3)
